# Evaluating Fundamentals of Vaginal Surgery for Use Among Obstetrics and Gynecology Residents in Accra, Ghana

**DOI:** 10.1007/s00192-025-06343-3

**Published:** 2025-09-25

**Authors:** Marie Bangura, Gabriel Ganyaglo, David Kupualor, Alex Boateng, Perez Sepenu, Isaac Koranteng, Emma R. Lawrence, Deborah Rooney, Payton Schmidt

**Affiliations:** 1 Department of Obstetrics and Gynecology, University of Michigan, 1500 E. Medical Center Dr. , Ann Arbor, MI 48109 USA; 2 Department of Obstetrics and Gynecology, Korle-Bu Teaching Hospital, Accra, Ghana; 3 Department of Learning Health Sciences, University of Michigan, Ann Arbor, USA

**Keywords:** Medical education, Surgical simulation, Vaginal surgery

## Abstract

**Introduction and Hypothesis:**

We aimed to assess obstetrics and gynecology (Ob/Gyn) resident experience and confidence with vaginal surgery and ability to perform basic vaginal surgery skills using a vaginal surgery simulator at a teaching hospital in Ghana. We present validity evidence and a region-specific proficiency score that can be used for training.

**Methods:**

In this cross-sectional study at a teaching hospital in Accra, Ghana, Ob/Gyn residents in a 3-year training program completed a survey on demographics, vaginal surgery exposure, and surgical confidence. Using the Fundamentals of Vaginal Surgery (FVS) simulation system, participants performed five timed tasks twice each. A region-specific proficiency score of ≥ 266 was used to assess skill proficiency.

**Results:**

Thirty-four participants were enrolled. Of the 22 who provided details about types of vaginal surgery they had performed, only two (9.1%) had completed a vaginal hysterectomy in the past 12 months. All participants described being “not at all confident” in performing vaginal hysterectomy and other vaginal surgeries and “somewhat or slightly confident” in their surgical skills. The median (IQR) overall normalized score for the FVS hands-on skills assessment was 57.5 (6.3, 113.8) for novice and 160.0 (95.8, 312.0) for experienced participants (*P* = 0.005). Five percent of novice and 28.6% of experienced participants achieved the proficiency score of ≥ 266.

**Conclusions:**

We present validity evidence and a region-specific proficiency score supporting the use of FVS in a Ghanaian training program. Limited experience in vaginal surgery, low confidence and performance scores highlight an opportunity to utilize this training system.

## Introduction

Minimally invasive hysterectomy, whether performed via a laparoscopic or vaginal approach, is associated with lower complication rates compared to open abdominal hysterectomy. Benefits include shorter hospital stay, faster recovery times, reduced postoperative pain, and lower risk of infection [1]. The vaginal route in particular is cost-effective and requires less specialized equipment compared to laparoscopy [2].

Despite key benefits to vaginal hysterectomy, global trends suggest that obstetrics and gynecology (Ob/Gyn) residents are getting less exposure to vaginal surgery and are less confident in performing these surgeries than previous generations [3]. This decline in experience has significant implications. Less experience with a surgical approach is correlated with higher complication rates when that approach is used, resulting in worse patient outcomes [4]. Moreover, this impact may be more profound in low- and middle-income countries (LMICs) where access to laparoscopy is limited, making vaginal surgery the primary minimally invasive option. Ensuring that Ob/Gyn residents receive adequate training in vaginal surgery is essential to maintaining access to safe, minimally invasive surgeries for patients—particularly in resource-limited settings.

In Ghana, although there is training in diagnostic laparoscopy, operative laparoscopy is not yet part of the required surgical curriculum for Ob/Gyn residents. As such, Ghana is a LMIC in which vaginal surgery is the primary minimally invasive surgical approach Ob/Gyns are exposed to during their training. Given this context, it is imperative that residents in Ghana are proficient in vaginal surgery to be able to safely offer this modality to their patients when feasible.

**Fig. 1:**
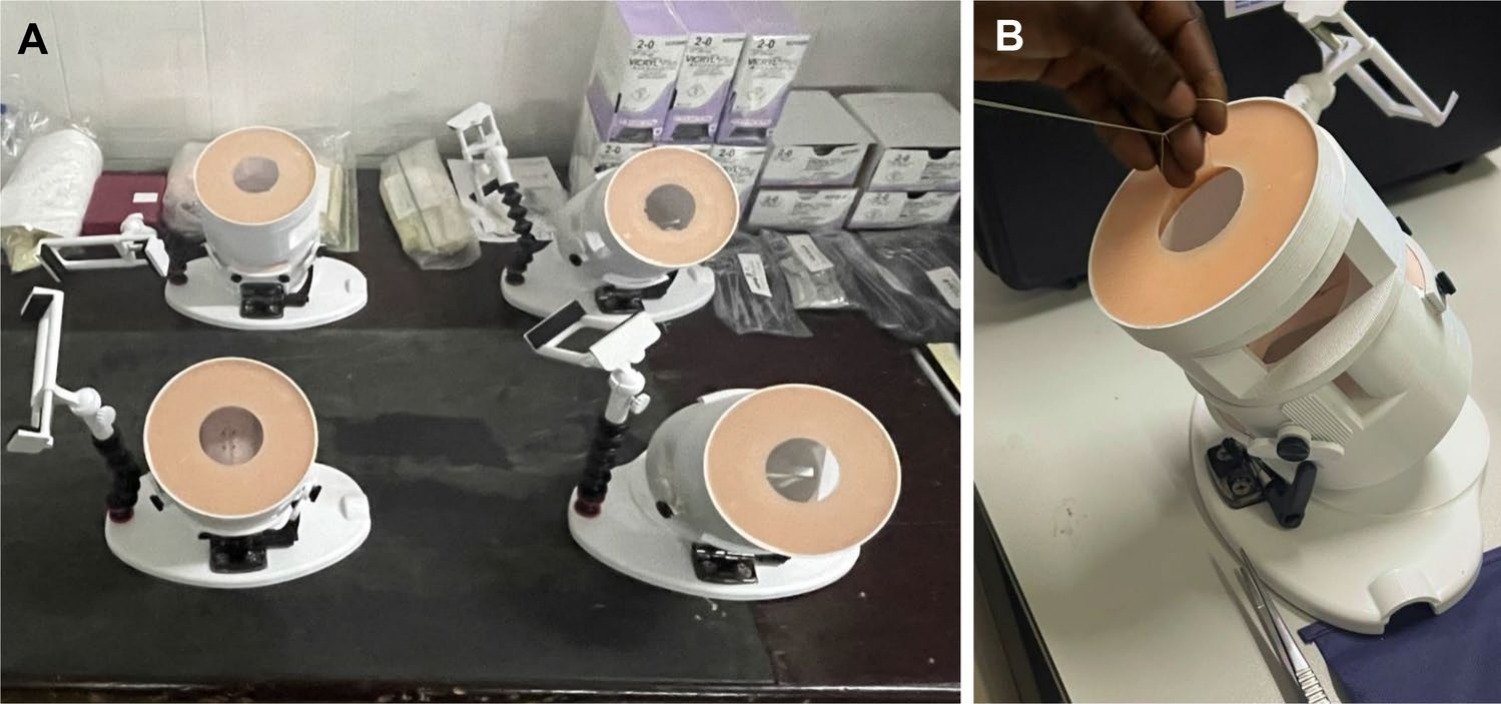
Fundamentals of Vaginal Surgery (FVS) simulation system **A** Several FVS trainers in the simulation lab at the teaching hospital **B** A participant using the FVS simulation system

Learning and mastering competency in basic surgical skills has been shown to improve intra-operative performance [5]. The Fundamentals of Vaginal Surgery (FVS) simulation system (Fig. 1) is a novel simulation system designed to teach and maintain the basic skills required to perform vaginal surgery. The FVS system includes a 3D-printed task trainer and disposable simulated inserts and has been previously validated in high-income countries [6]. Creating a simulation curriculum to improve basic vaginal surgical skills could be a way to optimize the learning opportunities residents have during training.

To implement a simulation curriculum such as FVS, the experiences of Ob/Gyn residents in Ghana with vaginal surgery and the baseline vaginal surgical skills must be understood. In this study, we aimed to assess Ob/Gyns resident experience with vaginal surgery, confidence in vaginal surgical skills, and their ability to perform basic vaginal surgery skills using a vaginal surgery simulator at a teaching hospital in Ghana. We also sought to collect evidence of validity and set a region-specific proficiency score for FVS.

## Material and Methods

This cross-sectional study was conducted in April 2023 at a large tertiary care hospital in Accra, Ghana, that is a referral center for management of complex obstetric and gynecologic conditions across southern Ghana. In 1989, it became the first teaching hospital in Ghana to offer an Ob/Gyn residency training program. The Ob/Gyn department is managed by five units, which are led by a senior consultant and staffed by other consultants and subspecialists, including maternal fetal medicine and oncology. Residents rotate with each unit and work with consultants to provide clinical and surgical care. In 2013, the hospital established a formal urogynecology training program. Initially, vaginal surgeries were primarily performed by a separate urogynecology unit. In 2023, urogynecologists were distributed across all units in the department to increase opportunities for trainees to participate in vaginal surgery.

The Ob/Gyn residency training program is completed over the course of 36 months.

In the first year, residents focus on foundational knowledge and surgical skills, with an emphasis on obstetric care. The second year requires 12 months of external rotations such as general surgery, urology, anesthesia, and neonatology to broaden clinical exposure. In the third year, residents manage more complex cases, with an emphasis on gynecologic surgery. There is variability in the number of residents enrolled from year to year, and therefore the exact distribution of trainees in each year was not available.

Participants in this study were Ob/Gyn residents currently enrolled in the residency program at the hospital. Residents were recruited by sharing information about the study at the daily Ob/Gyn department morning meeting, through the Ob/Gyn trainee text communication forums, and via the departmental email listserv. Of the 92 residents in the residency program at the time of the study, 34 (37.0%) enrolled in the study. The second-year residents were on external rotations, and the third-year residents were preparing for exams, which may have limited their availability to participate in this study. Written informed consent was obtained from all participants. Institutional review board (IRB) exemption was obtained from the lead author’s home institution and IRB approval was obtained from the study hospital.

Participants first completed an 8-item survey that collected demographic information: age, gender, year in training, and handedness. Next, they were asked to select which, if any, vaginal surgeries such as vaginal hysterectomy and posterior colporrhaphy they had performed in the past 12 months. Participants reported their level of confidence in performing these surgeries, as well as in basic vaginal surgery skills such as knot tying, suturing, and pedicle ligation, on a 6-point Likert scale (0 = not sure, 1 = not at all confident, 2 = slightly confident, 3 = somewhat confident, 4 = fairly confident, 5 = extremely confident).

After completing the survey, participants were introduced to the Fundamentals of Vaginal Surgery (FVS) simulation system (Fig. 1) [6]. There are six tasks a user can perform with the model: (1) one-handed knot tying, (2) two-handed knot tying, (3) running suturing, (4) plication suturing, (5) Heaney transfixion pedicle ligation, and (6) free-pedicle ligation. Prior to performing the task, the user watches a standardized instructional video (available at https://pfrg.smugmug.com/Fundamentals-of-Vaginal-Surgery) demonstrating how to perform each task. Users were timed; there was a cutoff time for completion of each task, with penalties to the user for errors or exceeding the allotted time. The completion time and penalties were subtracted from the cutoff time for each task. Exceeding the allotted time resulted in a score of zero for that task. Each task was performed three times, with the best score assigned as the overall score for that task. Each task score was then normalized by dividing the individual’s score by the maximum score achieved by a senior (third-year) resident for that task, then multiplying by 100 (normalized score = (individual’s score)/(maximum score achieved by resident) × 100). The final overall score was calculated by adding the scores of each task. The FVS pilot study established 400 as the proficiency score in the United States for the six tasks assessed with the model [6]. 

On the basis of feedback from faculty and the time allotted for participants to be excused from clinical duties to participate in the study, alterations were made in the FVS protocol. First, the running suturing tasks were removed from the skills assessment. Next, participants performed each task twice instead of three times. Finally, a new proficiency score was calculated on the basis of the median overall score on the five tasks for urogynecology fellows at the study hospital, as has been done in other basic skills programs [7]. The urogynecology fellows first complete an internship, followed by years of independent practice and Ob/Gyn residency. During their 3 years of training, fellows perform about eight vaginal cases per month, many of which are completed independently. As such, their performance is an appropriate benchmark for resident proficiency in vaginal surgery. The score used to assess proficiency on the five tasks (rather than six) evaluated in our study was 266 [6].

Performance was observed and scored by one of four Ob/Gyn fellows who had previously received training as evaluators on the FVS simulation system. After the conclusion of the skills assessment, participants completed a post-assessment survey, which asked whether each task in the skills assessment should be included in future simulation training.

IBM SPSS (version 29.0) was used to perform the statistical analysis. The data was not normally distributed, so median and interquartile ranges (IQR) were used to descriptively summarize the data. Mann–Whitney U test was used to compare differences in median performance measures after stratifying participants by level of experience. Novice participants were defined as first-year residents. Experienced participants were defined as second- or third-year residents. Since second-year residents had more surgical experience than first year-residents, they were included as experienced participants. The Medical Education Research Study Quality Instrument (MERSQI) score was generated to assess the quality of this study.

## Results

Demographic characteristics of the participants are presented in Table 1. Thirty-four residents participated: 20 (58.8%) first-year, 4 (11.8%) second-year, and 10 (27.8%) third-year. The participants had a median age of 35, were mostly male (73.5%), and primarily right-handed (97.1%).

**Table 1:** Participant characteristics (*N* = 34)

Characteristic	*n,* (%) or median (range)
Age, years	35 (33–37)
Gender	
Male	9 (29.5)
Female	25 (73.5)
Handedness	
Left	1 (2.9)
Right	33 (97.1)
Year in training	
First	20 (58.8)
Second	4 (11.8)
Third	10 (27.8)

**Fig. 2:**
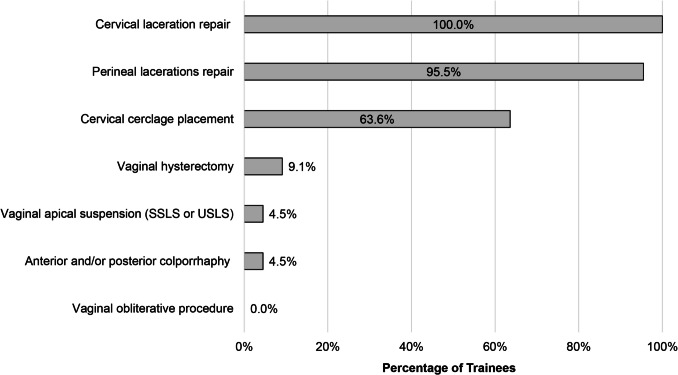
Participant self-reported experience performing vaginal surgery in the past 12 months. *SSLS* sacrospinous ligament suspension; *USLS* uterosacral ligament suspension

While the majority of participants (97.1%, 33/34) reported having participated in at least one vaginal surgery in the past 12 months, this experience was largely limited to obstetric procedures rather than those for gynecologic indications. Among the 34 participants, 22 provided details about the specific types of vaginal surgeries they had performed (Fig. 2). Of these participants (100%, 22/22) had performed cervical laceration repairs, 95.5% (21/22) had performed perineal laceration repairs, and 63.6% (14/22) had performed cervical cerclage placement. In contrast, only 9.1% (2/22) of participants had completed a vaginal hysterectomy and 4.5% (1/22) had performed pelvic floor-related surgeries in the past 12 months.

**Fig. 3:**
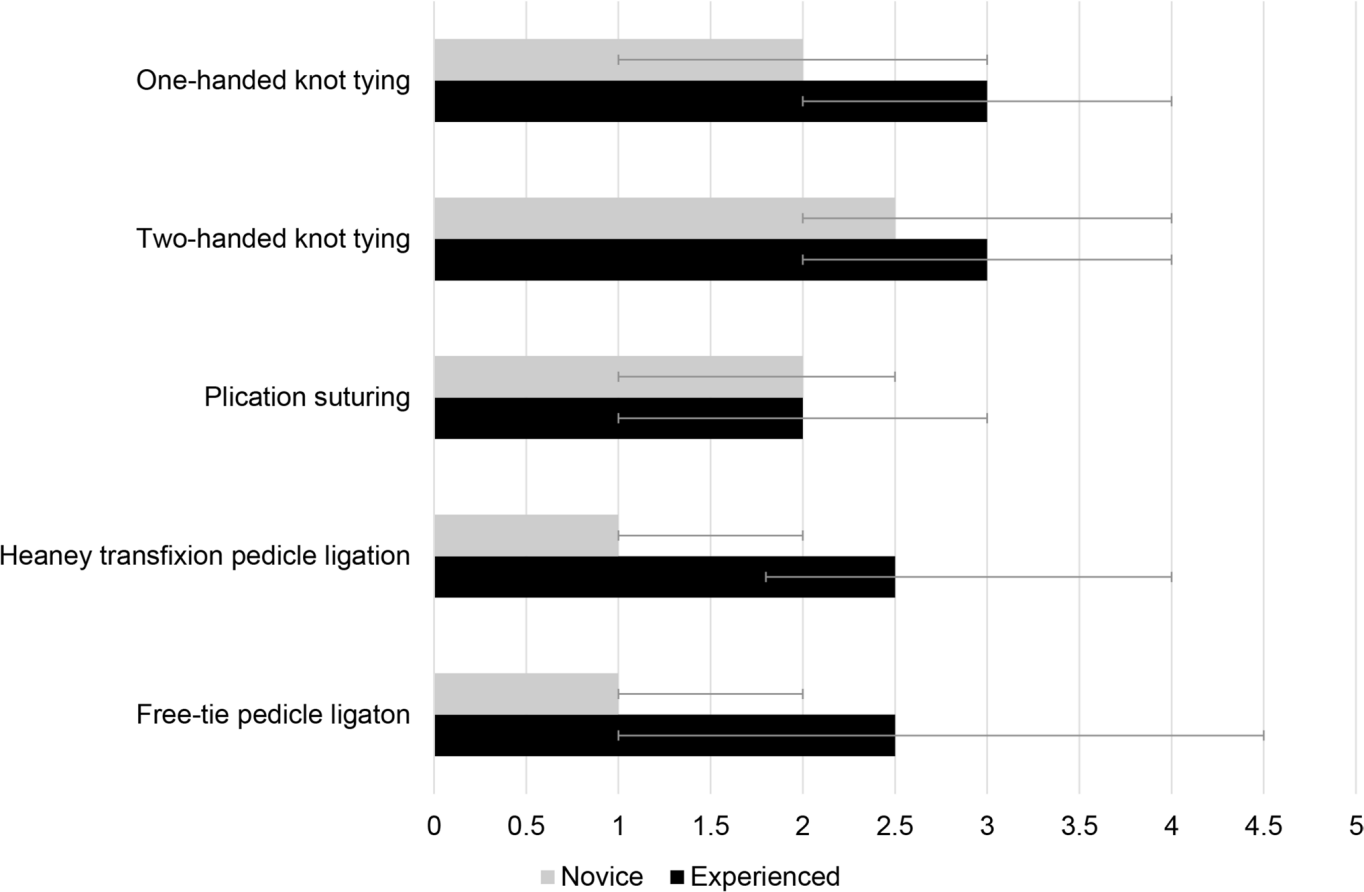
Confidence rating for surgical skills. Rating represented as median (IQR). Likert scale: 0 = not sure, 1 = not at all confident, 2 = slightly confident, 3 = somewhat confident, 4 = fairly confident, 5 = extremely confident

Confidence in performing basic vaginal surgical skills was overall low (Fig. 3). Novice participants described the most confidence in performing two-handed knot tying, with a median (IQR) confidence rating of 2.5 (2, 4)—“slightly to somewhat confident.” They were the least confident in performing pedicle ligations, with a median rating of 1 (1, 2)—“not at all confident”—for both Heaney transfixion pedicle ligation and free pedicle ligation. Experienced participants described the most confidence in performing one- and two-handed knot tying, with a median rating of 3 (2, 4)—“somewhat confident”—for both skills. Experienced participants were the least confident in plication suturing, with a median rating of 2 (1, 3)—“slightly confident.”

Similarly, confidence in performing vaginal surgery was low. The median confidence level among both novice and experienced participants was 1—“not at all confident”—in performing vaginal hysterectomy, anterior and posterior colporrhaphy, apical suspension, and Colpocleisis (data not shown).

**Fig. 4:**
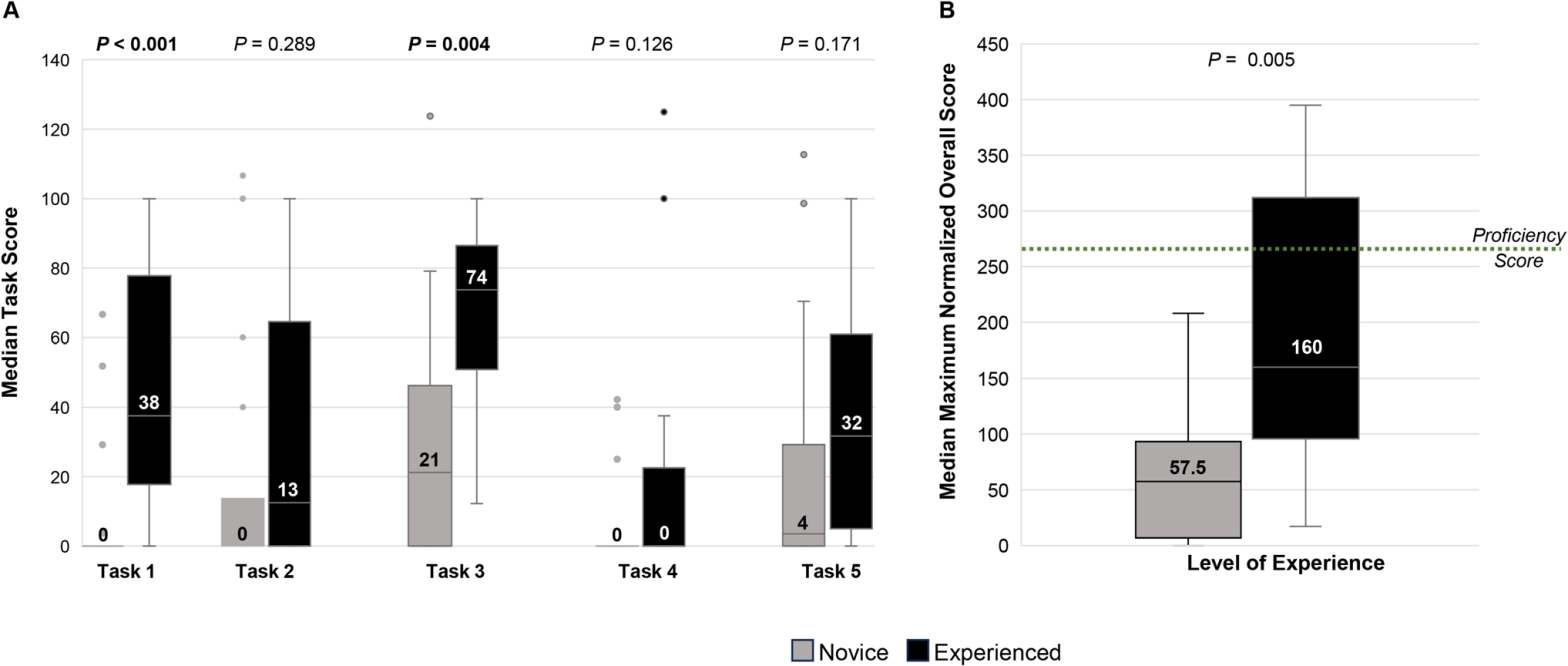
Scores for novice compared to experienced participants **A** Median scores for each task. Error bars show the range of scores for each task. **B** Maximum normalized overall score. Error bars show the range of overall scores

Hands-on assessment using the FVS simulation system demonstrated that baseline surgical skills scores were generally low. The median score for novice participants was 0 for task 1 (one-handed knot tying) and task 4 (Haney transfixion pedicle ligation), indicating the majority of novice participants did not complete each task within the cutoff time (Fig. 4A). Experienced participants consistently scored higher than novice participants on all five tasks; however, the difference was only statistically significant for task 1, one-handed knot tying (median score 0 vs 38 for novice and experienced participants respectively, *P* < 0.001) and task 3, plication suturing (median score 21 vs 74 for novice and experienced participants respectively, *P* = 0.004) (Fig. 4A). The proficiency score of ≥ 266 was the goal median overall normalized score for participants. Novice participants received a median score of 57.5 (6.3, 113.8) compared to 160.0 (95.8, 312.0) for experienced participants, *P* = 0.005 (Fig. 4B). While one novice participant achieved the proficiency score of ≥ 266, four (28.5%) experienced participants did. In the post-assessment survey, all participants either “agreed” or “strongly agreed” that all five tasks should be included in simulation training in the future.

Table 2 summarizes evidence of validity present in this study using the Messick validity framework [8]. The MERSQI score for this study is 12, demonstrating appropriate methodologic quality [9].

**Table 2:** Validity evidence

Validity source	Evidence
Content	Established through expert review; FVS tasks reflect essential vaginal surgical skills [6] FVS was previously validated at a US institution, now modified to meet the needs of the local institution
Response process validity	Participants watched standardized videos; trained fellows scored the performance A structured scoring system was used
Internal structure validity	Internal consistency among task scores (Cronbach’s α = 0.781)
Relationships to other variables	Scores are compared across experience level; experienced participants scored higher than novice participants Novice participants, with minimal prior vaginal surgical experience, had lower scores (as expected)
Consequences	The region-specific proficiency score was used to assess proficiency on basic vaginal surgery skills; scores ≥ 266 were deemed proficient based on expert performance

## Discussion

This study provides insight into the vaginal surgery experiences and skills among Ob/Gyn residents in Ghana. While most residents had participated in a vaginal surgery within the past 12 months, these experiences were largely limited to obstetric procedures. As a result, residents reported low confidence in performing vaginal skills and surgeries for gynecologic indications. These findings were consistent across all years of training, with experienced residents also reporting low confidence. A region-specific proficiency score was used to assess the residents. The low performance scores among novice residents were expected, given their early stage in training and the emphasis on obstetric skills in the first year of residency at this program. Moreover, despite describing limited experience with vaginal surgery, four experienced participants (28.6%) achieved the proficiency score of ≥ 266. This suggests that during their training, residents do acquire skills that translate to vaginal surgery. However, there is a need to increase dedicated training in vaginal surgical skills. This study provides evidence of validity for the use of FVS in this training setting.

Our findings are consistent with a global concern about limited training in vaginal surgery among Ob/Gyn residents [10–12]. Our study highlights the need to improve exposure to gynecologic vaginal surgeries and have dedicated practice with vaginal surgical skills for residents. This is particularly crucial in Ghana, where vaginal surgery remains the main minimally invasive gynecologic surgical approach available for patients at this time. Training residents in vaginal surgery is essential to ensure patients have access to this surgical approach and the benefits associated with minimally invasive surgery.

The lack of exposure to vaginal surgery for residents is not limited to our study site. Though there is limited published data, low case volumes for vaginal hysterectomies have been reported at other teaching hospitals in West Africa. In a prospective study conducted from January 2018–December 2019 at Komfo Anokye Teaching Hospital in Kumasi, Ghana, 87% of all benign hysterectomies were performed via open abdominal surgery, 13% were performed vaginally, and none were performed using laparoscopy [13]. Similarly, a teaching hospital in Nigeria reported 87.5% of hysterectomies were performed abdominally and 12.5% were performed vaginally [14]. Other teaching hospitals in Nigeria report rates as high as 21.8% for vaginal hysterectomy, but still perform the majority of cases open since those institutions do not have access to operative laparoscopy [15]. Collectively, this underscores the challenge of inadequate exposure to vaginal surgery, which may limit the ability of residents to develop proficiency in essential vaginal surgery skills. In this study, we created a region-specific proficiency score that can be utilized in developing skills in the simulation lab early in training to optimize intraoperative vaginal surgery experience that occurs later in training.

In our study, the FVS simulator was able to distinguish between novice and experienced residents, demonstrating its potential as a tool for both teaching and assessing improvements in surgical skills. Simulation-based training can improve both skills and confidence by providing opportunities for deliberate practice and feedback, which are necessary for skill acquisition [16]. This concept was reaffirmed by findings from a systematic review and meta-analysis that highlighted the effectiveness of both high-fidelity and low-fidelity surgical simulation in enhancing technical skills compared to traditional surgical instruction for gynecologic surgery [17]. More recently, a randomized controlled trial investigated the impact of procedure-specific simulation training on vaginal surgery skills among residents. The trial found that residents who underwent training with low-fidelity vaginal surgery models showed significant improvements in operative competence, self-confidence, and procedural knowledge compared to usual training methods [18]. Additionally, other surgical subspecialties have shown that building proficiency in the simulation lab can result in improved intraoperative experience [19]. Incorporating vaginal surgery simulation using the FVS simulation system into resident education could help improve confidence and surgical skills.

Our study has several notable strengths. It is the first study conducted in Ghana to assess vaginal surgical training. We identified a need for increased exposure to vaginal surgeries among Ob/Gyn residents, which can guide efforts to improve this area of their surgical education. Additionally, the study used FVS, a validated simulation tool, and demonstrated its utility in assessing surgical skills proficiency. The inclusion of residents from each year of training allowed us to observe that surgical skills generally improve with experience, as expected. However, most third-year residents did not achieve the proficiency score, highlighting the need for more focused training in vaginal surgery. Finally, we set a region-specific proficiency score and collected evidence of validity that supports the use of the FVS simulation system in this training setting.

In spite of these strengths, there are some limitations to our study. It was conducted at a single institution, which may not fully capture the variability in training experiences across different teaching hospitals in Ghana. Although 92 residents were enrolled in the residency program, we could not verify how many were in each year or who were on external rotations. These uncertainties were reflected in our low study participation of 34%, and it is likely that not all of the 92 residents were available on site at the time of this study. Moreover, the most experienced residents (third years) who should have had the most exposure to vaginal surgery, were underrepresented in our study. Thus, the performance score may not fully represent the performance of all third-year residents. Future studies are needed to capture additional data on performance across the training spectrum.

In conclusion, our study is consistent with global concerns about decreased experience with and confidence in vaginal surgery among Ob/Gyn residents. At this teaching hospital in Ghana, our study reveals that Ob/Gyn residents have limited exposure to vaginal surgery, which is reflected in their low confidence levels and performance scores. This suggests a need to improve vaginal surgical education. The FVS simulator could be used as a valuable tool for both teaching and assessing surgical skills. Integrating vaginal surgery simulation training into the residency curriculum as early as the first year could better prepare residents for the surgical opportunities they do have in the future, ensuring they can maximize learning and skill development. Future research should evaluate more validity evidence for the FVS scoring system within this clinical region, evaluate skills acquisition with the FVS system, explore the long-term impact of such training, and determine the feasibility of implementing similar programs in other LMICs.

## Data Availability

The data collected for this study are available from the corresponding author on reasonable request.

## Declarations

### Previous Presentations

Consortium of Universities for Global Health, Los Angeles, CA, April 2024. Society of Gynecologic Surgeons, Orlando, FL, March 2024.
